# The Increasing Incidence of Neuroendocrine Neoplasms Worldwide: Current Knowledge and Open Issues

**DOI:** 10.3390/jcm11133794

**Published:** 2022-06-30

**Authors:** Roberta Elisa Rossi, Sara Massironi

**Affiliations:** 1 Gastroenterology and Endoscopy Unit, IRCCS Humanitas Research Hospital, Via Manzoni 56, Rozzano, 20089 Milan, Italy; 2 Division of Gastroenterology, European Reference Network on Hepatological Diseases (ERN RARE-LIVER), San Gerardo Hospital, Department of Medicine and Surgery, University of Milano-Bicocca, 20900 Monza, Italy; sara.massironi@libero.it

Neuroendocrine neoplasms (NENs) include a heterogeneous group of tumors derived from neuroendocrine cells, most commonly arising from the gastro-entero-pancreatic (GEP) and bronchopulmonary tracts [1,2]. Although NENs have traditionally been considered rare tumors, their incidence has greatly increased over recent decades, partially due to better disease knowledge, the spread of large-scale screening campaigns, and an improvement in diagnostic tools, particularly endoscopy and nuclear medicine. Moreover, the increased incidence was particularly relevant in the stomach (15-fold) and rectum (9-fold), as a reflection of the increased use of endoscopic procedures, including the colorectal screening campaign [3]. Data from the Surveillance, Epidemiology, and End Results Program (SEER) database of the US National Cancer Institute suggested that NEN were more prevalent than hepatobiliary, esophageal, and pancreatic adenocarcinomas combined [4]. According to a recent population-based study that included a total of 43,751 patients, the age-adjusted incidence rate of GEP-NENs increased 6.4-fold from 1975 to 2015; among site groups, the incidence of rectal NENs increased most significantly, whereas as for stage and grade, the incidence increased particularly for the most localized GEP-NENs and G1 neoplasms [5]. Furthermore, reflecting both the rising incidence and the indolent nature of NENs, the 20-year limited-duration prevalence of GEP-NENs increased significantly from 0.00138% in 1996 to 0.03917% in 2015 [5]. Of note, even if the increase in incidence characterized all sites and stages, it was markedly greater for the localized stage, as a consequence of an improved diagnosis of asymptomatic, early stage disease, whilst the proportion of patients with metastatic disease has remained constant over time [6].

Historically, NENs have proven difficult to diagnose, given their nonspecific presentation, which can overlap with other clinical conditions. In this regard, the most typical example is represented by patients with small bowel NENs who often present with vague symptoms and are erroneously diagnosed with inflammatory bowel disease and/or irritable bowel syndrome by general gastroenterologists. As a matter of fact, NENs are often diagnosed when they are already metastatic with a consequent dismal prognosis. However, the improvements in widely used imaging modalities together with endoscopy and nuclear medicine have led to the increased detection of early stage, asymptomatic diseases, which in turn, are characterized by more favorable outcomes [7].

It is, therefore, important to keep in mind that, besides the well-known improvement in diagnostic tools over the years, the increased incidence might be partially dependent on better disease awareness. Furthermore, even if the reportedly improved outcomes with a consequent rise in the prevalence of NENs might be partially attributable to stage migration due to the increase in early stage diagnoses [6], the improvements in systemic therapy have, indeed, also contributed.

Another interesting aspect to be considered as a possible explanation for the registered increase in NENs’ incidence is the risk factor exposure; however, only a few factors with inconclusive results have been identified so far. A family history of any cancer, smoking, and gall-bladder disease/cholecystectomy have been reported to be associated with ∼a 1.5-fold increased risk of developing small bowel NENs [8]. Defined risk factors for pancreatic NENs include a family history of multiple endocrine neoplasia type 1 (MEN 1), which confers a 30–80% lifetime risk for developing these neoplasms, smoking, alcohol, and diabetes mellitus [9]. However, solid evidence is lacking regarding the actual role of these risk factors in the development of NENs.

Overall, a significant increase in both the incidence and the prevalence of NENs has been registered over the last decades, particularly in the United States and Canada; however, although to a lesser extent, this positive trend has also been observed in Europe as well as in Asia, thus highlighting the true increase in NENs. The reported differences according to geographic areas might be partially due to heterogeneity of data capture by different registries or to underlying biologic factors, environmental factors, and health care patterns, although a clear-cut explanation is still lacking.

Several improvements have been registered in the diagnostic setting. First, the diagnosis of small bowel NENs, which has always been extremely challenging because of both the lack of specific symptoms at presentation and the poor accessibility of the distal small bowel, has hugely improved with the advent of capsule endoscopy and double-balloon enteroscopy, although solid evidence regarding their actual role in the neuroendocrine setting is still scarce [10]. Ultrasound endoscopy (EUS) represents the diagnostic gold standard for pancreatic NENs and the technique of choice for the loco-regional staging of gastric, duodenal, and rectal NENs [11]. According to the latest European Neuroendocrine Tumor Society (ENETS) Consensus guidelines, EUS was proven to be the most accurate diagnostic technique in pancreatic NEN detection, leading to an up-to-94% sensitivity [12]. Furthermore, advanced EUS techniques may be helpful in the differential diagnosis of pancreatic NENs and the choice of the best-suited treatment. Regarding nuclear medicine, the sensitivity of 68Ga-SSA PET/CT for NEN is >90%, with specificity ranging between 92% and 98% [13]. It plays a pivotal role in the detection of the primary tumor being able to detect even small lesions (i.e., 5 mm) and in the identification of mesenteric lymph nodes and/or local tumor extension to determine the most appropriate surgical approach; it is also necessary for disease staging, being accurate in the detection of distant metastases, particularly bone metastases, in which the presence significantly affects patient’s prognosis. Another aspect to be explored is the role of biomarkers in the diagnosis and the follow-up of NENs. Chromogranin A, the most commonly known neuroendocrine general biomarker, is characterized by a low-specificity and might be more useful in the follow-up rather than in the diagnostic setting as a screening tool due to the suboptimal specificity [14,15,16]. On the other hand, the NETest represents the first successful attempt to provide a multianalyte signature in the blood that has clinical utility in the management of this composite disease. In an ideal world, the societies and guidelines should promote the introduction of novel technology utilizing real-time mathematical analysis of transcriptome-based disease assessment [17].

In terms of therapeutic options, the management of some tumors has changed over the years as there is, indeed, a trend toward less invasive approaches. The most typical example is represented by pancreatic NENs. Considering that, in the most recent decade, we observed a dramatic increase in the diagnosis of small, incidentally discovered, non-functioning pancreatic NENs [3,5], and a clear relationship between the tumor diameter and the risk of malignancy and recurrence has been reported [18], ENET guidelines started to recommend a “wait and see” approach for small asymptomatic non-functioning pancreatic NENs [12], and a European trial is currently ongoing [19]. Furthermore, endoscopic ablative technologies may also be utilized in patients with pancreatic NENs not suitable for surgery or who refused the surgical approach. Duodenal NENs are heterogeneous, still poorly understood tumors as, despite clear differences, their management is treated along with either gastric or, if functioning, pancreatic NENs. Endoscopic resection is increasingly performed instead of surgery. However, duodenal NENs are characterized by a highly variable prognosis and, despite the small size, can be metastatic in up to 55% of cases, either at diagnosis or thereafter [20]; additionally, considered that conventional imaging has a poor detection rate for loco-regional nodes and micro-metastases in the presurgical setting, the choice of local conservative approaches, including endoscopy or local surgical excision, should be carefully balanced and discussed by a multidisciplinary team and EUS should always be included in the preoperative phase for a more accurate local staging [21]. Rectal NENs have shown a dramatic increase in their incidence, as they are more and more frequently incidentally found during screening colonoscopies. A conservative approach (i.e., endoscopic resection) is recommended for well-differentiated rectal NENs smaller than 10 mm, whereas the best management of tumors between 10 and 20 mm, in which the metastatic risk is intermediate and the endoscopic treatment can be challenging, is still unclear [22]. Several medical therapies for NENs are currently available, including somatostatin analogs, targeted agents, and chemotherapy; a specific mention should be reserved for Peptide Receptor Radiotherapy (PRRT), which based on the recent randomized controlled trial [23], has become an established treatment for malignant metastatic GEP-NENs. As a future perspective, trials focusing on immunotherapy are ongoing for patients with NENs, but no clear-cut data are currently available.

Although NENs are becoming less rare tumors and knowledge of these neoplasms is increasing, given their biological heterogeneity, there is an urgent need for standardized guidelines for the proper management of these neoplasms, which should always be referred to tertiary referral centers. Even if many reports and guidelines regarding NEN management are available and new treatment options for clinical management have been developed, many patients are still referred to specialists with no or low expertise in the neuroendocrine field with a consequent diagnostic delay. It is, in fact, not uncommon that a NEN patient is managed in non-NEN specialized or dedicated structures, where the management of the patient relies on the vision of a single doctor, or on the choice of the patient and/or family to seek second opinions elsewhere. To avoid such scenarios, a multidisciplinary approach and a network between referral centers are necessary to offer every patient the best approach from both a diagnostic and a therapeutic view. It is mandatory to develop novel research strategies to better define diagnostic and therapeutic algorithms, particularly for some specific subgroups of poorly known tumors, including duodenal NENs and functioning tumors. The close cooperation between peripheral and referral centers, and the creation of international disease registries need to be encouraged. The advances in molecular and genetic sciences may be helpful for the application of novel approaches, including neoadjuvant or adjuvant targeted options.

## Data Availability

No new data were created or analyzed in this study. Data sharing is not applicable to this article.
